# Decision boxes for clinicians to support evidence-based practice and shared decision making: the user experience

**DOI:** 10.1186/1748-5908-7-72

**Published:** 2012-08-03

**Authors:** Anik Giguere, France Légaré, Roland Grad, Pierre Pluye, R Brian Haynes, Michel Cauchon, François Rousseau, Juliana Alvarez Argote, Michel Labrecque

**Affiliations:** 1 Health Information Research Unit, Department of Clinical Epidemiology and Biostatistics, McMaster University, CRL-139 1280 Main Street, West Hamilton, ON L8S 4 K1, Canada; 2 Research Center of the CHUQ, Saint-Francois d’Assise Hospital, 10 rue de l’Espinay, D6-730, Quebec City, (QC), G1L 3 L5, Canada; 3 Department of Family Medicine, McGill University, 515-517 Pine Avenue West, Montreal, (QC), H2W lS4, Canada; 4 Department of Clinical Epidemiology and Biostatistics and Department of Medicine, DeGroote School of Medicine, McMaster University, 1280 Main Street West CRL-125, Hamilton, ON, L8S 4 K1, Canada; 5 Department of Family Medicine and Emergency Medicine, University Laval, 1050 avenue de la Médecine, Room#4617, Quebec, (QC), G1V 0A6, Canada; 6 Universidad del Valle, Calle 4B No. 36 – 00 Edificio 100 1er piso, Cali, Colombia

**Keywords:** Evidence-based medicine, User experience, Risk communication, Usability, Patient-centered care, Counselling, Clinical topic summary, Decision support, Knowledge translation, Communication design

## Abstract

**Background:**

This project engages patients and physicians in the development of Decision Boxes, short clinical topic summaries covering medical questions that have no single best answer. Decision Boxes aim to prepare the clinician to communicate the risks and benefits of the available options to the patient so they can make an informed decision together.

**Methods:**

Seven researchers (including four practicing family physicians) selected 10 clinical topics relevant to primary care practice through a Delphi survey. We then developed two one-page prototypes on two of these topics: prostate cancer screening with the prostate-specific antigen test, and prenatal screening for trisomy 21 with the serum integrated test. We presented the prototypes to purposeful samples of family physicians distributed in two focus groups, and patients distributed in four focus groups. We used the User Experience Honeycomb to explore barriers and facilitators to the communication design used in Decision Boxes. All discussions were transcribed, and three researchers proceeded to thematic content analysis of the transcriptions. The coding scheme was first developed from the Honeycomb’s seven themes (valuable, usable, credible, useful, desirable, accessible, and findable), and included new themes suggested by the data. Prototypes were modified in light of our findings.

**Results:**

Three rounds were necessary for a majority of researchers to select 10 clinical topics. Fifteen physicians and 33 patients participated in the focus groups. Following analyses, three sections were added to the Decision Boxes: introduction, patient counseling, and references. The information was spread to two pages to try to make the Decision Boxes less busy and improve users’ first impression. To try to improve credibility, we gave more visibility to the research institutions involved in development. A statement on the boxes’ purpose and a flow chart representing the shared decision-making process were added with the intent of clarifying the tool’s purpose. Information about the risks and benefits according to risk levels was added to the Decision Boxes, to try to ease the adaptation of the information to individual patients.

**Conclusion:**

Results will guide the development of the eight remaining Decision Boxes. A future study will evaluate the effect of Decision Boxes on the integration of evidence-based and shared decision making principles in clinical practice.

## Introduction

Resources for finding medical evidence have evolved greatly in the past few years. Searches take less time and results are more relevant than ever before. For many clinical questions, however, even the best available evidence does not always produce a single best answer. In some cases, the scientific evidence about outcomes is insufficient; in others, proof of benefit is more or less counter-balanced by proof of harm. In 2007, *Clinical Evidence* classified 51% of treatments as having insufficient evidence and 7% of treatments as tradeoffs between benefits and harms [1].

Where two or more medically acceptable options exist, the choice should depend on the patient’s circumstances, values, and preferences [2]. Values and preferences refer to patients’ perspectives, beliefs, expectations, and goals for life and health, and more broadly to the processes patients use to consider the options and their relative benefits, harms, costs, and inconveniences [3]. To make an informed choice, patients thus need access to the best available information, presented in a format that makes it easy for them to make a decision consistent with their values and preferences [4].

Since 1992, the field of shared decision making has mainly focused on developing and evaluating patient decision aids: interventions designed to translate information more directly to patients and to help them better clarify their values [5]. Most patient decision aids are designed so that patients can work through them on their own. Studies show that printed, electronic, and audiovisual patient decision aids help increase patients’ knowledge, feeling of being adequately informed, and participation [5], and reduce the overuse of screening or treatment options not clearly associated with health benefits for all [6]. The research also shows, however, that while patients want information about their medical condition and treatment, they do not necessarily wish to be responsible for deciding on treatment [7]. In other words, patient decision aids can only go so far: patients want their healthcare provider’s input on their care. Other than training and continuing medical education programs for healthcare professionals [8], relatively less efforts have focused on how to foster a culture where clinicians embrace shared decision making as a clinical skill [9].

In this article, we present a tool designed for clinicians that aims to improve the participation of both patient and clinician in the decision-making process. This tool, called the Decision Box, is intended to help the clinician recognize that a decision needs to be shared with the patient, prepares the clinician to communicate evidence-based information to the patient, and assists the clinician in seeking patient’s values and preferences regarding the decision to be made. The Decision Box is a short clinical summary [10,11] that integrates the best available evidence from studies and syntheses to provide quantitative information on management options. It is specialized to cover medical questions that have no single best answer. More than a summary, though, it is framed in a way to help the user weigh the risks and benefits of all options in light of the patient’s individual health status. It also offers guidance on the shared decision-making process.

Our objective was to develop Decision Box prototypes, test them with patients and clinicians, and try to improve them by addressing the barriers identified during user testing with regards to the communication design. More specifically, this paper presents the process used to select clinical topics for 10 Decision Boxes, the evaluation of users’ experience of the tool, and the pre-test of a questionnaire that we will use in a future implementation study.

## Methods

This project was approved by the research ethics committees of the Centre de Recherche du Centre Hospitalier Universitaire de Quebec and McGill University.

### Selection of clinical topics for the decision boxes

Using a Delphi survey described elsewhere [12], a panel of seven of the researchers involved in this project (including four practicing family physicians) selected 10 clinical topics they perceived as relevant to primary care practice. Because of our interest for the translation of genetic innovations to the population, we initially instructed the panelists to select two genomic topics among the 10, but at the second round we changed our instructions and asked them to select three instead. The panel was instructed to select topics that did not have a single best choice: *i.e.*, the decision addressed should enclose scientific uncertainty about the outcome or balance of benefits and harms [5]. Panelists were asked to propose additional topics after the first round of the survey. At each round, we retained the topics that were chosen by all panelists, and removed the topics that were chosen by three panelists or less. The survey was stopped after three rounds.

### Development of the decision box prototypes

To develop the Decision Box prototypes, we chose two topics that were more interesting to clinicians (selected early in the Delphi), that targeted different populations to maximize the diversity of participating patients, and that would be easier to explain because patients were generally familiar with them. We thus chose ‘Prenatal screening for the detection of trisomy 21’, and then had a choice between ‘Colorectal cancer screening with fecal occult blood test’ and ‘Prostate cancer screening with the prostate-specific antigen (PSA) test’. We chose PSA because of the controversy around this test.

We planned the documents so they would respond to the learning objectives of the training program (Table 1). We developed a first version of the Decision Box on PSA testing, based on the communication design of the Drug Facts boxes [13] and on research on risk communication (described in [12]). At this stage, the document consisted in a simple two-column black and white table with text and colour graphics. It was presented to the members of the Canada Research Chair in Implementation of Shared Decision Making, in Quebec (Canada), who grave their opinion on the elements that should be removed and those that should be kept, together with general comments on the tool. We modified this version and submitted it to a graphic designer who chose the graphical display. We then produced the second prototype on prenatal screening directly with the graphic designer. Presentations of the tools to experts in shared decision making (SDM), knowledge translation, and genomics at scientific meetings led to four more versions before the design proved satisfactory.

**Table 1:** Specifications for the decision box independent learning program

**General objective:** To use shared decision-making principles to involve patients in the decisions regarding each of the clinical questions covered.	
**Specific learning objectives**	**Instructional activities**
· To describe the available options for each clinical question;	· Reception of a series of Decision boxes, at regular intervals, by email;
· To describe the specificity and sensitivity of the test (only for screening procedures);	
· To describe the probabilities of risks and benefits of the available options for each clinical question;	· Reading of the Decision boxes;
	· Assessment of each Decision box using a web-questionnaire;
· To revise the pros and cons of each of the available option for each clinical decision;	· Reading of the additional resources on patient counselling provided on the website;
· To judge the quality of the best available data for each clinical decision;	· Viewing the tutorial on the Internet;
· To list the questions to identify patients’ decision making needs;	· Using the information provided in the Decision box in practice when there is an opportunity.
· To list additional resources available to patients to address their decision making needs.	

**Figure 1:**
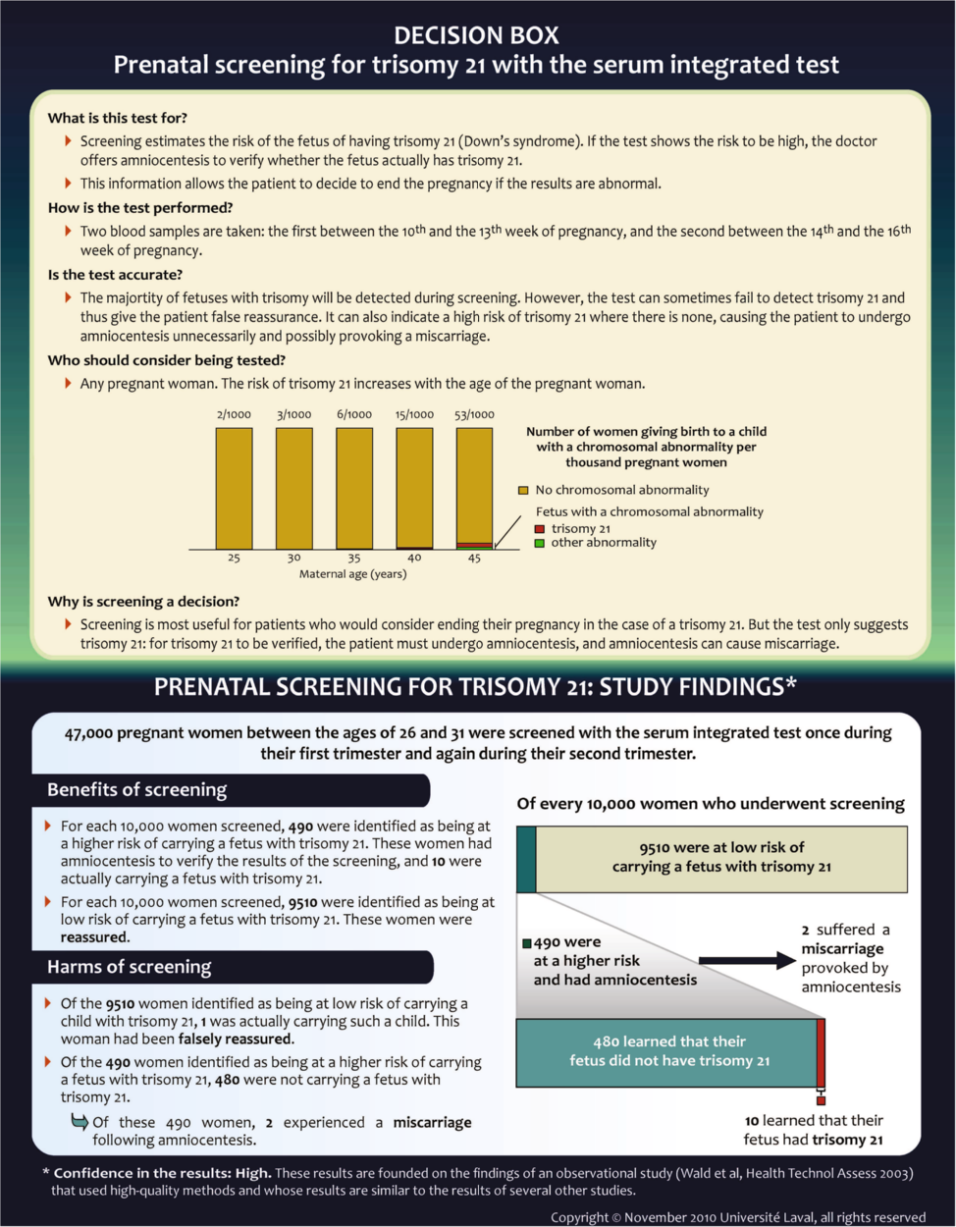
The Decision Box prototype on prenatal screening for trisomy 21 (BEFORE evaluation).

**Figure 2:**
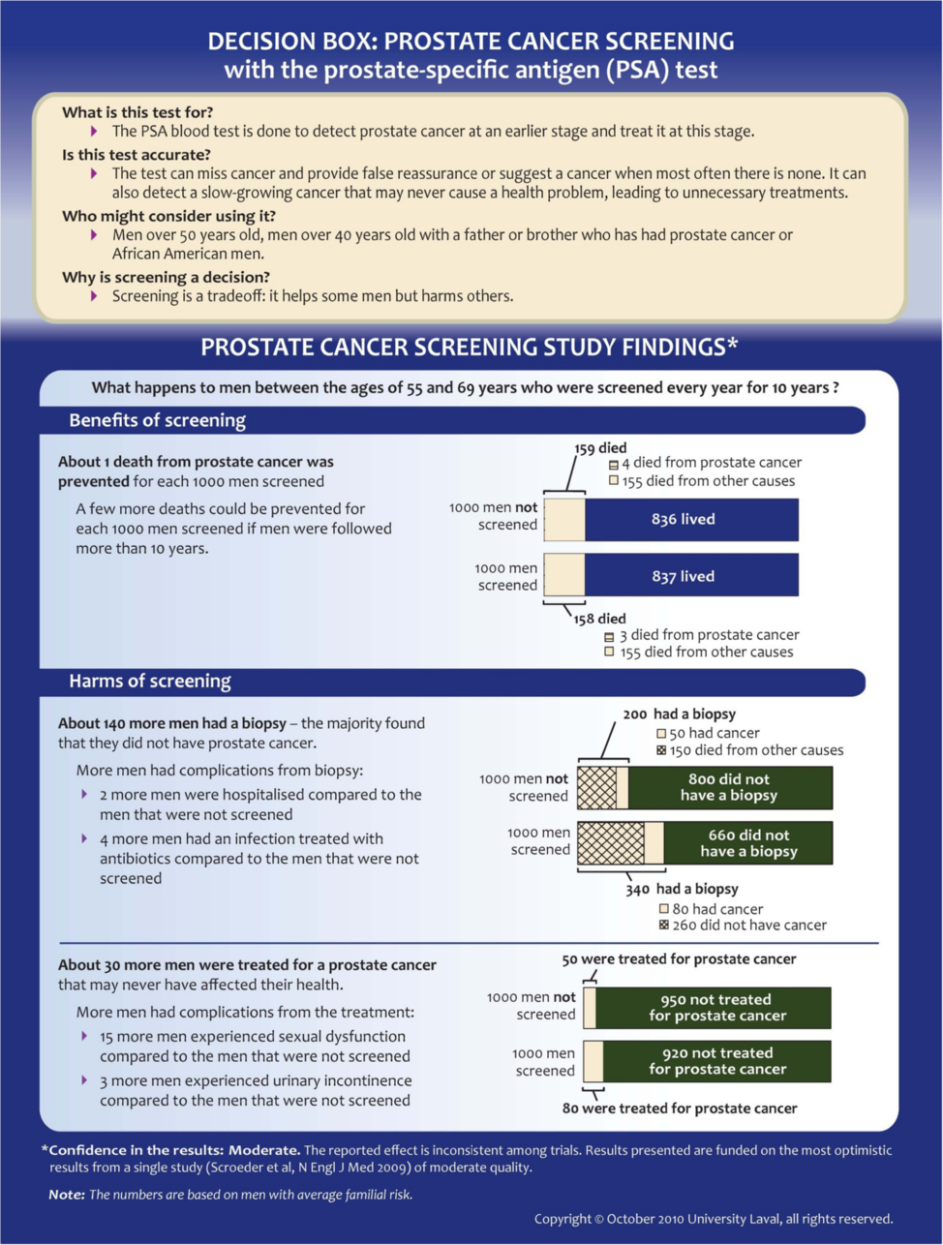
The Decision Box prototypes on prostate cancer screening (BEFORE evaluation).

Each prototype was written in both French and English, and divided into three sections (Figures 1 and 2). At the top, the ‘Presentation of the intervention to patients’ section described the intervention for which a decision was required, in straightforward lay language. This section broadly described the accuracy of the test, the population for which intervention might be appropriate, and clarified the decision to be made. The ‘Study Findings’ section presented the results of a single study that we considered to be the most relevant to present population or ‘average risks’ of benefits and harms of the intervention and to have the highest strength of evidence. This section first described the study (the study population and the length of follow-up) in a single sentence and then combined narration, graphics, and numbers to outline the study’s findings about the intervention’s benefits and harms. We used a few additional studies to present important elements that were not covered by the main study, for example, the proportion of miscarriages following amniocentesis, but to simplify the document we did not describe the studies themselves, and we did not give references to these studies. At the bottom, a ‘Confidence In The Results’ section gave publication information about the main study on which the ‘Study Findings’ section was based and made a statement about the study’s quality and its consistency with other published studies on the same topic. This assessment was adapted from the methodology of the Grading of Recommendations Assessment, Development and Evaluation (GRADE) Working Group [14].

A graphic designer produced the prototypes as one-page color documents. The main title of each prototype was ‘Decision Box’; the subtitle stated the intervention. The endorsement of each prototype by Laval University and the date of the last update were placed in small letters at the bottom.

### Users’ experience of the decision box prototypes

#### Participant inclusion criteria and the recruitment process

To explore users’ perceptions of the communication design used in the prototypes and to seek suggestions for improvement, we conducted two focus groups with family physicians and four focus groups with patients. Using their professional networks, two members of the research team (ML and RG) recruited practicing family physicians from the Family Medicine Units in Quebec and Montreal in Canada. Patients were recruited from these two sites and met criteria for participation if they were: men between 45 and 75 years old, or women between 20 and 40 years old who wanted to have a child, were pregnant, or were already mothers. Participants received a monetary compensation for their participation. The clinics’ support staff distributed information sheets about the study to eligible patients. Interested patients then contacted the research team to participate.

### Focus groups

We presented the two Decision Box prototypes to two groups of family physicians: first, a French-speaking group and second, an English-speaking group. We presented the same prototypes to a purposeful sample of patients who agreed to participate and who qualified for the study (in other words, we presented the prototype on prostate cancer screening to male volunteers and the prototype on prenatal screening to female volunteers). We used a maximum variation strategy to populate the samples, which we segregated by mother tongue. In this way, we constituted four focus groups of patients: French-speaking men, French-speaking women, English-speaking men, and English-speaking women.

We conducted all interviews at the clinics where participants were recruited. We used a semi-structured interview guide to explore participants’ experience of the communication design used in Decision Boxes based on Peter Morville’s User Experience Honeycomb [15]. More precisely, physician interviews explored the tool’s value in preparing them to communicate scientific information to patients and helping patients make informed, value-based decisions. The patient interviews explored how patients felt about their physician reading the Decision Box before their clinical encounter to better prepare for their visit, and to ask them whether the Box contained all the information they needed to make a decision.

The focus groups were moderated by two experienced interviewers: one for the French-speaking groups and one for the English-speaking groups. One moderator held a master’s degree in anthropology and the other a master’s degree in library and information science. Both were research professionals at the time of the study. Two observers (AG and either PP, ML or RG) took notes on the process and content of the discussions. One observer (AG) was a postdoctoral fellow, one was a researcher (PP), and the others (RG and ML) were family physicians and colleagues of the participating physicians. No physician was present during interviews of one of his/her own patients. One observer (AG) was present at all focus group discussions, to ensure consistency in the approach. All discussions were audiotaped and professionally transcribed.

### Questionnaires

At the beginning of each focus group, we collected demographic data from all of the participants and questioned them about their health history regarding the topic addressed in the Decision Box. After the focus group, the patients were administered the Decisional Conflict Scale [16]. Following the focus groups, family physicians pre-tested a self-administered questionnaire to be used in a larger study on the implementation of the Decision Boxes (Additional file 1). This questionnaire evaluated the respondents’ perceptions of the Decision Box on PSA testing for prostate cancer screening. The questionnaire measured physicians’ interest in the clinical topic using a visual analog scale that ranged from 0 (no interest) to 10 (deep interest). It also comprised the information sub-scale of the Decisional Conflict Scale [16], the Information Assessment Method [17], a scale based on the Theory of Planned Behaviour that evaluated physicians’ intention to use in their practice what they had learned from the Decision Box to help their patients make an informed decision [18], and a scale based on the Technology Acceptance Model (TAM2) that evaluated physicians’ perceptions of the usefulness and ease of use of the Decision Box [19].

### Analysis

One researcher (AG) and two research professionals performed a thematic qualitative data analysis of the content of focus group discussions following a hybrid deductive/inductive approach [20]. This analysis identified barriers and facilitators to the participants’ experience with the prototypes. The deductive analysis searched for attributes related to the seven facets of the User Experience Honeycomb [15]: valuable, usable, credible, useful, desirable, accessible, and findable. The inductive analysis integrated new themes mentioned by participants. First, to assess whether the User Experience Honeycomb applied and to explore possible sub-themes, the researcher and the two research professionals separately went through the same portion of one of the focus group transcripts and noted any attributes related to the Honeycomb. The three coders then compared their results and came to a consensus on the themes and sub-themes mentioned in this transcript sample. Next, they noted these themes and sub-themes in a manual of codes, labelling and defining them as well. The transcripts were entered as project documents into specialized software (NVivo 9, QSR International, Cambridge, MA, USA), and the codes developed for the manual were entered as nodes. One research professional then applied these codes to the English interviews while the other applied them to the French interviews to identify meaningful units of text. The first author (AG) then read all transcripts and reviewed the codes applied by the research professionals to the six interviews to ensure completeness and appropriateness of the code manual, and consistency of approach. Again, any modifications to the predetermined code manual were discussed among the three coders until consensus was reached.

We modified the Decision Boxes to take into account users’ comments. We also performed descriptive statistical analyses of the answers to the questionnaire.

## Results

### Selection of clinical topics

For genomic topics, two rounds were necessary for a majority of panelists to select the same three clinical topics (Table 2). For the other topics, the final selection was achieved after the third round. In round one, one panelist selected a single genomic topic instead of two as instructed, and nine of the other topics instead of eight. At the second round, one panelist selected six topics instead of the five as instructed. Only prostate cancer screening and colorectal cancer screening with the fecal occult blood test were unanimously selected during the first round of the survey.

**Table 2:** Clinical topics and number of panelists selecting each topic at each survey round (*: indicates the round at which the topic was selected)

**a) Genomic topics**		**No of panelists**	
		**Round 1**	**Round 2**
· Screening for BRCA1 or BRCA2 gene mutations to evaluate the risk of breast and ovarian cancer		5	7*
· Newborn screening for sickle cell anemiaa		0	-
· Newborn screening for MCAD (medium chain acyl-CoA dehydrogenase) deficiency		0	-
· Hereditary colorectal cancer screening in individuals with high risks of colorectal and endometrial cancers		1	4*
· Screening for hereditary hemochromatosis in patients with abnormal ferritin level or abnormal transferrin saturation or in patients with family members with hemochromatosis		0	-
· Prenatal screening for the detection of Down’s syndrome, Trisomy 18, and open neural tube defects		5	7*
· Genotype testing for patients initiating warfarin treatment		1	3
· Screening for hypertrophic cardiomyopathy (HCM) in individuals with clinical features or a family history of HCM		0	-
· Testing for CDKN2A and CDK4 gene mutations to evaluate the risk of melanoma and the predisposition for pancreatic cancer		1	0
· Genetic testing for factor V Leiden to identify those at an increased risk for venous thromboembolism (VTE).		0	-
**Additional topics proposed by the panelists at first round**			
· Screening for hereditary prostate cancer		-	-
· Screening for MEN predisposing mutations		-	0
**b)Other topics**	**No of panelists**		
	**Round 1**	**Round 2**	**Round 3**
· Colorectal cancer screening with faecal occult blood test	7*	-	-
· Prostate cancer screening with the prostate-specific antigen (PSA) test	7*	-	-
· Abdominal aortic aneurysm screening with abdominal ultrasound	1	-	-
· Treatment of mild to moderate depression with selective serotonin reuptake inhibitors (SSRIs) or St. John’s wort	2	-	-
· Treatment of Alzheimer’s disease with cholinesterase inhibitors	5	4	5*
· Antibiotic treatment for patients with exacerbated chronic obstructive pulmonary disease (COPD)	4	4	4
· Intensive glycemic control in type 2 diabetes	4	4	5*
· Prevention of strokes with antithrombotic therapy in patients with atrial fibrillation	3	-	-
· Prevention of cardiovascular events with antihypertensive drug in patients over 60 years old presenting with essential hypertension	4	2	-
· Prevention of vascular diseases with acetylsalicylic acid (ASA)	6	6	7*
· Prevention of osteoporotic fractures with bisphosphonates (alendronate, risedronate, etidronate) in postmenopausal women	6	4	7*
· Primary prevention of cardiovascular disease with statins in persons with cardiovascular risk factors	5	4	7*
· Prevention of cervical cancer with the vaccine against the human papillomavirus (HPV)	3	-	-
**Additional topics proposed by the panelists at first round**			
· Antibiotics versus nasal steroids for the treatment of acute sinusitis in adults	-	1	-
· Early palliative care for patients with metastatic non–small-cell lung cancer	-	1	-
· Omega-3 for mild to moderate depression	-	1	-
· Delayed versus immediate antibiotic prescriptions for acute uncomplicated urinary tract infection in women	-	0	-
· Prevention of prostate cancer with 5 alpha-reductase inhibitors (finasteride and dutasteride)	-	2	-
· Breast cancer screening for women age 40 to 50 with no risk factors	-	1	-
· Prevention of breast cancer with tamoxifen	-	2	-

### Users’ experience of the decision Box prototypes

#### Participants’ characteristics

Eighteen of 35 (51%) eligible physicians agreed to participate, and 15 attended the interviews—all participants stayed until the end. All physicians who did not agree to participate stated they were not free on the date of the focus group. Seven physicians participated in the French group interview and eight in the English interview; 73% were women. Most were between 30 and 60 years old (median = 40 years old) and had practiced medicine for 5 to 37 years (median = 13 years). The four groups of patients totalled 33 participants. Within groups of women and groups of men, levels of education, employment status, and age were similar, regardless of language; but these characteristics differed between groups of men and women (Table 3).

**Table 3:** Characteristics of participating patients in each focus group

	**Women**		**Men**	
	**French**	**English**	**French**	**English**
**n**	9	8	9	7
**Median age (min : max)**	30 (20 : 38)	30 (24 : 38)	54 (46 : 62)	51 (47 : 66)
**Employment status (n)**				
Employed full-time	6	6	3	5
Employed part-time	0	1	1	2
Unemployed and seeking employment	0	1	1	0
Unemployed and not seeking employment	0	0	0	0
Retired	0	0	3	0
Other	3^*^	0	1^∀^	0
**Highest education level (n)∴**				
No high school	0	0	0	0
Some high school - did not graduate	0	0	0	1
High school degree or certificate of equivalency	0	0	3	0
Some college – did not graduate	0	0	0	0
College degree	2	1	1	2
Some university – did not graduate	1	1	5	0
University degree	6	6	0	3

* 1 preventive withdrawal because of pregnancy and 2 students.

∀ absence from work as a result of sickness.

One response is missing in the English-speaking men’s group.

Among the 17 women participants, 10 had been pregnant at least once and none had a child with trisomy 21. Seven went through prenatal testing for trisomy 21, and none received a positive result. One had an amniocentesis, and her fetus was not diagnosed with trisomy 21. Of the participating women, 10 had received information on prenatal screening before the interview and mentioned several sources of information (sometimes more than one source): eight mentioned their doctor, their medical clinic or the hospital; three mentioned the internet; and some mentioned a university course, a specialised prenatal private clinic, friends and family, and books. Of the 16 men who participated, nine had been screened for prostate cancer at least once, four had received a positive result following screening, and three had had a biopsy. In two of the three, the biopsy had revealed prostate cancer, for which they were treated. Eight of the male participants had received information on prostate cancer screening before the interview. As sources of information on the cancer, seven mentioned their doctor, their medical clinic, or the hospital, two mentioned an advertisement or the television, one medical publications, and one family members.

### Focus groups

The focus groups with physicians lasted about one hour and 45 minutes and those with patients lasted about one hour. Patients’ and physicians’ perceptions of the Decision Boxes mostly concurred, although the physicians discussed more the data, whereas patients discussed more the shared decision-making process.

**Figure 3:**
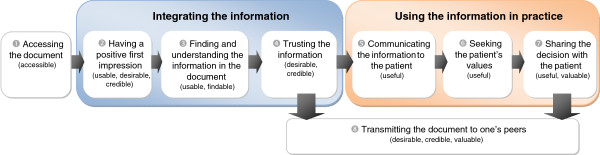
**Steps of the users’ experience of an evidence-based shared decision-making support tool over time.** Correspondence between each step and facets of the model used to develop the interview guide (*i.e.*, Morville’s User Experience Honeycomb) are shown in parenthesis.

The User Experience Honeycomb used to develop the interview guide describes seven facets (or qualities) of an individual’s experience of a product. During coding, we felt that the user’s experience of a Decision Box would be best represented as a process, because the facets at play change from the time when clinicians access the Decision Box to the time when they use it in their practice. Following our analysis, we propose eight successive steps to users’ experience of an evidence-based shared decision-making support tool over time, and we describe which facets of the users’ experience are at play at each of these steps (Figure 3). The eight proposed steps are successive: for the document to accomplish its purpose (here, to assist the physician to share a decision with the patient), the user must go through each step in sequence, one after the other.

When analyzing the interviews, we coded barriers and facilitators to users’ experience of Decision Boxes at each step. These results are detailed in the Table 3a to 3g, and we can draw a few more general observations.

### Accessing the information

Multiple communication channels were proposed by participants to facilitate access to Decision Boxes. Even if we specified to participants that Decision Boxes were developed to be used primarily before the clinical encounter, some still proposed to use them during the clinical encounter and suggested that printed format or mobile applications might then be more useful than the Internet.

### Integrating the information

Most of the interviews’ discussions concerned the value of the communication design to integrating the information. Understandability, the format of graphics and missing information were the factors most often reported as barriers to the ‘Finding and understanding the information’ step (Table 4, section c). Comments on the understandability of the information mostly referred to how the presentation of the information allowed the user to pinpoint the risks or the benefits of the presented options. Participants generally found the proposed bar charts to be complex, and many suggested not using any, or using alternate representations such as flow charts or little men (icon array). Among the data found to be missing by participants, they reported that alternative screening tests should be described. Concerning the format of numbers, one-half the groups mentioned that presenting percentages would be helpful. Some barriers to trusting the information were more often reported for the prostate cancer prototype, namely subjectivity and information differing from what participants already knows.

**Table 4:** Factors related to users’ experience of the Decision Box and frequency of interviews where they were mentioned (Men = participant from the men’s group; Wo = participant from the women’s group, MD = participant from the physicians’ group; FR = French-speaking group; En = English-speaking group)

**Factor**	**Illustrative participant response (group)**	No of groups in which the factor was mentioned as a*	
		**Facilitator**	**Barrier**
a) Accessing the Decision Box *			
- As printed document/not as printed document	It has some important information that I could just have handy instead of looking on the Internet at that moment. (MD-En)	2	2
- In an electronic format	You can use something electronic if you’re sitting in front of a computer and you show your patient the screen. (MD-En)	2	
- As mobile version	Now that it exists as application for our pocket computers, we do not carry this with us all around. (MD-Fr)	2	
- Multi-channel distribution	I actually like having something physical, especially if it’s going to be used to counsel patients. I like an Internet link to educate myself. (MD-En)	1	
**b) Having a positive first impression**			
Appropriate/inappropriate titles	I was expecting pros, cons. This is kind of what I went through with the pregnancy with Cedric. I had to go through understanding the pros and the cons and debating whether I wanted to do it or not. (Wo-En)	3	3
Credibility/lack of credibility	it’s going to have more credibility when they know that there is no drug company. (MD-En)	3	1
Too much text or too many numbers, too dense	Too busy, too busy. (MD-En)		3
Sound, explicit methodology	It needs to come from an organization that can say, “Look, we did the research, and this is what we know in 2011. We will update the box in 2013 at the latest.” (MD-Fr)	3	
Simplicity - length	It’s like a cheat sheet. On a cheat sheet, you would squeeze everything, every tiny little drop that you could. This is a cheat sheet and it’s all in one page. I’m happy. (MD-En)	3	
Good color choice	I think, color wise, it works fine because there is nothing that is offensive here. You don’t have a lot of red which may be more scary than other colors so, color wise I think it works well. (Men-En)	3	
Including/not including an introduction	It has a good presentation section that says [to the physician], this document is about this and that, and after it’s described to you, then you will “get it” (…) It is not like having it on my desk without preparation. (MD-Fr)	2	2
Information differs from participants’ prior knowledge	At present, so many patients are screened. Why…what’s the story, why are specialists proposing such things, you know. How did we get to this point? (MD-En)		2
Time contraints	I thought it was going to be, like, an algorithm, that I can follow. And this is not. I would not have time to read it for sure.(MD-En)		1
**c) Finding and understanding the information in the Decision Box**			
Information understandable/not understandable	I think it’s clear. I mean, it gives you… it makes a statement. And then, it shows… it gives you the explanation of that statement. And if you read both of them, you realize that first statement is just being explained on this right hand side. I think it’s pretty clear. (Men-En)	5	6
Appropriate/inappropriate format of graphics	When I first looked at it, I said: “Oh, the graphics are quite… not complicated but there’s lots of stuff on it”. (MD-En)	3	6
Missing information	We are often doing the rectal touch together with PSA testing…They only describe the PSA here…I would have preferred that they discuss rectal touch too. (MD-Fr)		6
Appropriate/inappropriate format of numbers	If you say your chance is .5%, well that sounds a lot better than saying, you know, 5 out of a thousand. (MD-En)	3	5
Good source of information	Could it be a good sheet for physicians that do not see pregnancies often, for residents? I think that yes. In these cases, there is interesting information, really substantiated.	5	
Develops critical thinking	Patients, too, learn that medicine is not perfect. We are in a high-technology era; everybody thinks that we can do anything, that we can know everything. It’s a widespread idea. This makes it possible to set the record straight for us and for patients. (MD-Fr)	4	
Allows conceptualization	It helps us conceptualize. (MD-Fr)	4	
Inappropriate language or style	The second sentence, what is the test for, it allows the patient to decide to end the pregnancy. It’s written negatively. It should be either to continue or to end the pregnancy. (Wo-En)		4
Data presented but not found	Talking about age, it says here that the risk of trisomy increases with age of the pregnant woman. It would be good to know by how much. (Wo-En)		4
Information too basic	When I first saw this, it seemed to me that every doctor should know this already. By heart, without even consulting it. In all honesty, it seems to me the information here is pretty basic. (Wo-En)		3
Bold important words and use more bullets	The other thing I would say is that, again, for the bottom part, the benefits of screening and the harms of screening, as for the previous, I think it would be more useful to have it a little bit more [text] bulleted and bold. (MD-En)	2	
Appropriate to have both words and graphics	It’s good that there is a graphic side and a text side, even if in one way, the same information is found in both places. There is information for people who learn best when they see something and information for those who like to read. (Wo-Fr)	1	
Objectivity/subjectivity	Somebody has a preconceived notion, who wrote this, that you should not, that it’s not a good idea to go through the screening. And they framed it in that way. (Men-En)	4	3
Confidence in results section improves credibility/undermines credibility	It says it’s a moderate, single study of moderate quality. So, you know, it’s not the best. (Men-En)	1	4
References not detailed enough	Is this study a knowledge synthesis, meaning that they took information from everywhere and synthesized it? Or is it a single study? (…) If they verified in 3-4 types of study sites, for me that counts more than a study that was performed in a single location. (Wo-Fr)		4
Information differs from what participant already knows	I am glad to know this side of the story, but I’d like to see the other side. Cancer prevention associations state that screening saves lives, but I don’t remember seeing any numbers. (MD-Fr)		4
Typos or mistakes	Optimistic results, I’ve never heard that expression. So, I was sort of already thrown off. I didn’t even get to “I like this part.” (MD-En)		3
Scientific quality of the tool	It is really important to have up-to-date results. (Wo-Fr)	3	
**e) Communicating the information to the patient**			
Synthesized and simplified information	It’s obvious that it can give doctors the words they need to explain things more simply to their patients. (Men-Fr)	6	
Easy/difficult to apply to individual patients	I’m 38 now. Can my doctor sit me down in 5 years and use these results to tell me, no, you don’t need to do a test, or yes, you need to do a test? I mean, it doesn’t apply. (Wo-En)	2	5
Information well-organized/Information does not flow	It’s nice how they’ve divided it. The top kind of allows them to position where their individual patient is in terms of where they are at, where their symptoms are and then the bottom section gives the broader scope of the research. (Wo-En)	4	4
Time required to use Decision Boxes would decrease with familiarity/time constraints	When the physician’s in his office, at the speed he sees me, I am not sure he’d start explaining this. (Men-Fr)	2	2
**f) Seeking the patient’s values**			
Lack of non-scientific information	On one hand, I like that it is neutral and factual, but at the same time, I wish there were a way to make it more human, to suggest reflection. (Wo-Fr)		5
Would be good to generate discussion	If I think it can help me discuss things with a patient. You know, if it can help having more arguments or easier language or other, new knowledge, I would keep it. (MD-En)	2	
Only scientific information is needed	I don’t think your doctor should question your values or your moral beliefs or your religion. It should be scientifically based. It shouldn’t open up that door. It’s personal. (Wo-En)		2
Doctor’s lack of communication skills	Even if everything is written as completely as it is here, [even if] the doctor has all the data, he can still come along and bang! be a real drip and say, “Here we go, here we go, now just go on home and think about it. Come back when you’re ready to tell me what you’ve decided.” (Wo-Fr)		2
Too complicated to be discussed with the patient	For me, in the harms [section], it makes a little more sense to have of the 1000 men screened pros and cons but not necessarily the other number. To have the “not screened” [number] makes the discussion much more complex. (MD-En)		2
**g) Finding and understanding the information in the Decision Box**			
Lack of non-scientific information	On one hand, I like that it is neutral and factual, but at the same time, I wish there were a way to make it more human, to suggest reflection. (Wo-Fr)		5
Would be good to generate discussion	If I think it can help me discuss things with a patient. You know, if it can help having more arguments or easier language or other, new knowledge, I would keep it. (MD-En)	2	
Only scientific information is needed	I don’t think your doctor should question your values or your moral beliefs or your religion. It should be scientifically based. It shouldn’t open up that door. It’s personal. (Wo-En)		2
Doctor’s lack of communication skills	Even if everything is written as completely as it is here, [even if] the doctor has all the data, he can still come along and bang! be a real drip and say, “Here we go, here we go, now just go on home and think about it. Come back when you’re ready to tell me what you’ve decided.” (Wo-Fr)		2
Too complicated to be discussed with the patient	For me, in the harms [section], it makes a little more sense to have of the 1000 men screened pros and cons but not necessarily the other number. To have the “not screened” [number] makes the discussion much more complex. (MD-En)		2
**h) Trusting the information**			
Having appreciated using it	When we discuss something among colleagues and I say: “Look, I used this in this way and it really helped me”. If I don’t use it, there’s not much chance that I will share it with my colleagues. (MD-Fr)	4	
Simplicity of the tool – length/Inappropriate size – too large	It’s simple. Like, I’m not going to hand them 15 pages. (MD-En)	1	1
Good source of new information	The fact that you said that it’s the best evidence we have, I find that interesting. (MD-En)	1	
Credibility of the source	Because if you guys were sending me this, a box of these, then I would say: “Oh look the College is endorsing me to use these Decision Boxes.” Well, o.k. I’m more likely to give them to a colleague. (MD-En)	1	
Clinically-based information	… But the idea that it’s clinically based…You give more credit to something like that. And you get more people to read it. (MD-En)	1	
Clear concept –easy to understand	If the concept is clear enough. If it is easy to understand. (MD-Fr)	1	
Difficult to apply to individual patients	I am used to talking about the triple-test. The only value added of a sheet is to cite numbers that I don’t keep in the top of my mind. People want to know their risk at 35 years old, their risk of this and that. I can’t apply this as it is. (MD-Fr)		1

*Only the 2 physician groups discussed this step.

### Using the information in practice

Concerning the tool’s usefulness in the clinical setting, there was a general perception that synthesized and simplified information facilitated the communication of information to patients (Table 4, section e). Yet, five groups reported difficulty applying the information to individual patients as a barrier to communicating the information. There was general agreement that a lack of non-scientific information was a barrier of the Decision Box to seek patient values (Table 4, section f) and that guidance to make a decision was lacking. Specific aspects of the clinical topics covered in the prototypes (PSA or prenatal testing) were reported to influence the sharing of decisions with patients. For the PSA test, the lack of evidence on which to base a decision and risks’ outweighing benefits were perceived as barriers to sharing the decision with patients (Table 4, section g). Having appreciated using the Decision Box was most often reported by physicians as a facilitator to sharing of the Decision Box with peers.

### Prototype modification

**Figure 4:**
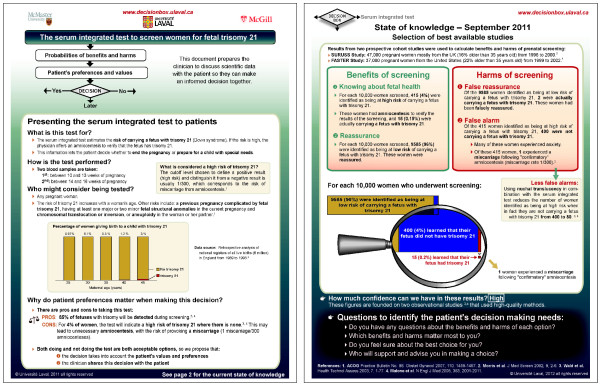
Decision Boxes on prenatal screening for trisomy 21 modified to reflect user experience testing (AFTER evaluation).

**Figure 5:**
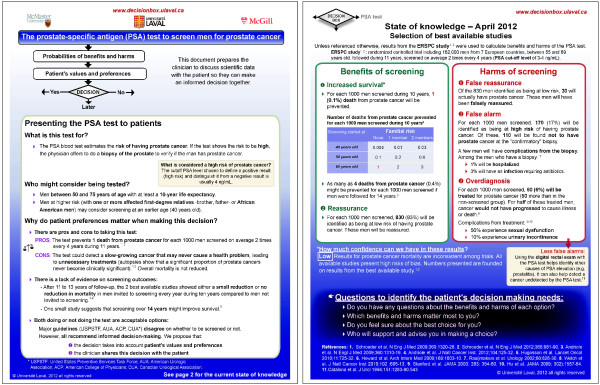
Decision Boxes on prostate cancer screening modified to reflect user experience testing (AFTER evaluation).

Following analyses, we modified the Decision Box prototypes to try to take the participants’ perceptions and suggestions into account (Figures 4 and 5). Redesigning of the prototypes emerge mostly from the analyses, as many solutions were found during the analysis phase, by discussion among the three coders. Potential solutions to the identified barriers were discussed again with the professionals and researchers of the Canada Research Chair in Implementation of Shared Decision Making. Redesigning of the graphic aspects was generally straightforward: the first author (AG) used the graphic design software herself, and integrated modifications to the prototypes as ideas were emerging. The graphic designer was involved more punctually to help resolve specific issues (for example, with the graphic on the Decision box on prenatal screening).

We first added three sections: an introduction-to-the-document section, a patient counseling section, and a reference section. The document was spread over two pages to make it look less busy and to try to improve first impressions. To try to improve the boxes’ credibility, we made more visible the names of the research institutions involved in developing the boxes. A statement on the boxes’ purpose and a flow chart representing the shared decision making process were added to clarify the Decision Box’s purpose. Where there was evidence, the presentation of risk factors and of benefits and harms of the intervention according to these factors became essential elements of the modified documents, because we thought that this would help physicians apply and communicate the information to individual patients. In the patient counseling section, we proposed three questions that physicians could ask patients to help clarify patients’ values and preferences and to guide the decision-making process. Last, hoping to clarify that the Decision boxes are based on the best available evidence, we added more references to studies and, when applicable, described the design used by the studies, the study participants, and the length of the intervention.

### Patients’ decisional conflict after the interview

After the interview, the patients’ mean Decisional Conflict Scale score was 25% (±SD 12%) and ranged from 2% to 44%, indicating low decisional conflict.

### Questionnaire for family physicians

Physicians’ mean interest in PSA testing for prostate cancer screening as a clinical topic was 7.9 ± 1.2 (SD) on a visual analog scale ranging from 0 (no interest) to 10 (deep interest). On the information subscale of the Decisional Conflict Scale, which ranged from 0 (feels extremely informed) to 100 (feels extremely uninformed), physicians gave a mean score of 25.6% ± 10.7 for the Decision Box on PSA testing for prostate cancer, indicating that they felt well-informed after reading it. Using the Information Assessment Method, all participants reported that the Decision Box on PSA testing had an impact on them or their practice. The most frequently reported type of cognitive impact was that it would remind them of something they already knew (93%). All physicians reported they would use this information for their patients, and the most frequently reported planned use was to resolve a doubt (60% of physicians). Eighty-seven percent of physicians expected the information to benefit their patients, with the most frequently reported expectation being that the information would make the patient more knowledgeable about health or healthcare (60%). Physicians’ intention to use in their practice what they learned from the document to help their patients make an informed decision averaged 5.4 ± 1.2 (SD) on a scale from 1 (strongly disagree) to 7 (strongly agree), indicating that they had the intention to use what they learned. On average, physicians perceived the Decision Box prototypes as being somewhat easy to use (4.2 ± 1.5) and useful (4.8 ± 1.0) on a scale ranging from 1 (strongly disagree) to 7 (strongly agree).

## Discussion

In this study, we explored facilitators and barriers to the communication design of two Decision Box prototypes by engaging users in their testing, and we modified these prototypes to try to minimize the influence of the observed barriers. Our findings improve understanding of the design of evidence-based shared decision-making support tools, which in turn will improve their value for end users.

### Accessing the documents

With 95% of Canadian physicians using electronic tools [21], the Internet might be the most efficient communication channel to deliver Decision Boxes to clinicians. However, participants suggested accessing Decision Boxes through multiple channels, such as printed documents, the internet, and mobile applications, especially for use during the clinical encounter. A website offering a one-click access to printable Decision Boxes may thus be useful in clinical encounters.

### Integrating the information

Newer information tools summarizing the current best evidence, such as Decision Boxes and synopses, respond to the widely acknowledged problem of information overload in healthcare. Brevity and lower density of information are especially critical to foster a positive first impression of such tools [22,23], and to improve users’ comprehension of the options [24]. Other factors are also at play during the first contact between the receiver and the message, such as the credibility of the source of information (expertise, trustworthiness) [25]. By clearly identifying three universities as the sources of Decision Boxes in the modified documents, we are building on the ‘reputed credibility’ that universities generally possess and on the ‘experienced credibility’ stemming from users’ academic experiences [26]. Participants also reported that knowledge of the tool development methodology would influence their first impressions of the Decision Boxes. Consequently, the website hosting the Decision Boxes will include a methodology subsection to describe the typical indicators needed to appraise clinical summaries, namely the methods used to search and update the literature, and to critically appraise the retrieved sources [10].

Our findings support another study that also reported understandability of the information as key factor of a positive user experience [27]. According to Rosenbaum [28], ‘understandability’ involves two separate dimensions: the users’ perception of their own understanding, which we explored in this study, as well as an objective measure of correct understanding that would need to be tested separately. Hoping to improve users’ perceptions of understandability, we modified the Decision Boxes to use percentages to convey probabilities whenever possible. Major organizations, such as the Cochrane Collaboration [29] and the International Patient Decision Aid Standards (IPDAS) Collaboration [30], have been recommending natural frequencies to present absolute risks. A recent randomized trial however compared adults’ understanding of five different numerical formats, and found the percent format had slightly higher comprehension overall [31]. Following participants’ comments on the understandability of the graphics used in the prototypes, we either removed them or simplified them, but we kept bar graphs because these have been reported to be readily understood and helpful [32]. The research literature is not clear on which graphs are most effective to communicate health risk [33,34].

Message attributes can also influence credibility [25], but at a later step that we named ‘Trusting the Information’. Participants reported objectivity and confidence in results as key message attributes that influenced their trust in the information. To foster perceptions of objectivity, negative and positive features of options should be presented equally to patients [30]. Objectivity might have been questioned more for the prostate cancer prototype, first because the size of the harms section was larger than that of the benefits, but also because its content differed from what most participants already knew. The theory of cognitive consistency proposes that information which is compatible with existing beliefs is the most likely to be accepted, and that which emphasizes the undesirable qualities of existing beliefs may be selectively avoided or ignored [35]. Comments on the ‘Confidence in Results’ section reveal that participants’ trust in Decision Boxes was influenced by the quality of evidence presented within. This supports findings from a study of the Cochrane Collaboration’s summary of findings table in which users also indicated that the table’s credibility was reduced when GRADE ratings were low [27,28].

### Using the information in practice

Following participants’ comments, we added information on the benefits and harms of the intervention according to individual patient risk, when available, to try to improve applicability of the Decision Boxes to practice. Risks can be personalized based on individual risk factors for a condition (such as age or family history), or it can be calculated using formulae derived from epidemiological data [36]. A review on the effectiveness of personalized risk communication in the context of screening showed little impact of this strategy to promote informed decision making [36], but another study reported primary care practitioners preferred personalized risks [37].

Participants mentioned the prototypes lacked non-scientific information, and this concurs with recommendations to base decisions not only on scientific evidence but also on patients’ values and preferences when two or more medically acceptable options exist [2]. To try to help patients clarify and express their preferences and values, the modified Decision Boxes proposed three questions for the clinician to ask patients. Participants also requested some guidance on the process of shared decision making and we are planning to provide such guidance in a tutorial on the website hosting Decision Boxes. The IPDAS Collaboration suggests that patient decision aids should provide a step-by-step method to make a decision or include tools like worksheets or lists of questions to use when discussing options with a health professional [30].

### Strengths and limitations

Because we recruited both English- and French-speaking participants, and both clinicians and patients, we were able to gather a large array of points of views reflecting some of the future users of Decision Boxes. We recruited a diversity of male patients, including two men who had been treated for prostate cancer. However, although we recruited one woman who had an amniocentesis following positive screening, we did not interview any woman who had had a child with trisomy 21.

Some biases may have affected the focus group interview. For example, the researchers’ presence may have biased responses towards more positive comments. Also, participants did not receive the documents in their usual context, and because they had more time to look at the documents, they may have underlined more problems than they would normally have been aware of. We tried to minimize this bias by setting the context at the beginning of the interviews.

Testing Decision Boxes on two different clinical topics was a strength of this study. Most the uncovered barriers are widely applicable to many topics because they describe communication design, rather than content issues, and so provide a good basis for developing future Decision Boxes. However, we did uncover some barriers/facilitators that were specific to topic in the present evaluation. Developers of new Decision Boxes should then consider testing with target users, an important step to uncover possible barriers specific to topic. For the eight remaining Decision Boxes, we are planning to further explore the influence of topic on users’ experience of the Decision Boxes.

Our interview guide and study design were useful mostly to explore barriers to integrating the information, because the participants really experienced these steps when reading the prototypes at the beginning of the focus groups. Because participants did not meet with patients after reading the Decision Boxes, the reported barriers to using this type of information in practice needs to be confirmed among a sample of clinicians who had the opportunity to use what they learned from the Decision Box with their patients. Our interpretation of participants’ comments led us to modify the prototypes to address the problems and limitations they perceived. We need to verify if our interpretation was right, and if the choices that we made to address the limitations of the prototypes truly improved the Decision boxes and did not generate new problems.

## Conclusions

We identified factors influencing the communication design of Decision Boxes that act when accessing them, when integrating the information presented within, and when using them in clinical practice. These factors will guide the development of the eight remaining Decision Boxes covering the other topics selected at the beginning of this project. In the next phase of this program, we will evaluate users’ perceptions of the Decision boxes that are to be developed following our interpretation to confirm that our modifications truly addressed the identified problems and did not generate new problems. We plan to use a mixed approach to collect users’ perceptions of the modified Decision boxes, using the questionnaire tested in this study and focus groups. In a longer term, we plan to evaluate the effect of Decision Boxes on the integration of evidence-based and SDM principles in clinical practice. We hypothesize that the implementation of Decision Boxes in clinical practice will prepare the physicians to better communicate the benefits and harms of the available options to their patients. Better communication will allow patients to become more involved in decisions concerning their health, and in turn lead to a more judicious use of current best evidence.

## Competing interests

The authors declare that they have no competing interests.

## Authors’ contributions

AG, ML, FL, RG, PP, RBH, MC, FR and FL contributed to the study plan, and to data collection via the Delphi survey. AG, ML, RG, PP contributed to data collection with focus groups. All authors contributed to the development of the Decision box prototypes. AG and ML contributed to data analysis. AG wrote the first draft of the manuscript. All authors reviewed the manuscript and approved its final version.

## Supplementary Material

Additional file 1 Questionnaire. Click here for file
