# Supplementary material for: Decision boxes for clinicians to support evidence-based practice and shared decision making: the user experience
Source: Implement Sci. 2012 Aug 3;7:72. doi: 10.1186/1748-5908-7-72 (PMC3533695; doi:10.1186/1748-5908-7-72)
Supplement: Additional file 1 — Questionnaire. [file 1748-5908-7-72-S1.doc]

**We would like to know your opinion of the document that you have just received.**

| 1. **Your date of birth:** | __________/_________/__________  (year / month / day) |
| --- | --- |
| 1. **Your gender:** | F  M |
| 1. **Year that you obtained your first medical license (even if obtained outside of Canada):** | ___________________ |
| 1. **What city do you practice in?** | ___________________ |
|  |  |

1. **How interested are you in the clinical subject discussed in this document?**

Draw a vertical line on the scale below to indicate your level of interest (example:). If you are completely uninterested, draw a line on the far **left**. If you are extremely interested, draw a line on the far **right.**

No interest

Great interest

***For the research team:***

*Time of distribution: __________________*

*Time of collection: ___________________*

1. **Now that I have read the information presented in this document...**

|  | **Strongly agree** | **Agree** | **Neither agree nor disagree** | **Disagree** | **Strongly disagree** |
| --- | --- | --- | --- | --- | --- |
| 1. I know the patient’s options |  |  |  |  |  |
| 1. I am aware of the benefits of each option. |  |  |  |  |  |
| 1. I am aware of the risks and side effects of each option. |  |  |  |  |  |

1. **Considering the information presented in this document, please answer the following questions:**

| **What impact does this information have?** | **Yes** | **Maybe** | **No** |
| --- | --- | --- | --- |
| My practice is (will be) changed and improved |  |  |  |
| I learned something new |  |  |  |
| I am motivated to learn more |  |  |  |
| The information confirmed that I have been doing the right thing |  |  |  |
| I am reassured |  |  |  |
| This information reminded me of something I already knew |  |  |  |
| I am not satisfied |  |  |  |
| There is a problem with this information |  |  |  |
| I do not agree with this information |  |  |  |
| I think that this information could be harmful |  |  |  |
| This information has absolutely no impact on me or my practice | | | |
| **Is this information relevant to one or more of your patients?** | | | |
| Very relevant | | | |
| Somewhat relevant | | | |
| Irrelevant | | | |

| **If this information is relevant to at least one of your patients, how do you plan to use it?** | **Yes** | **Maybe** | **No** |
| --- | --- | --- | --- |
| I will not use the information for one of my patients |  |  |  |
| I will use the information… |  |  |  |
| … to think about the patient |  |  |  |
| …to continue to interact with the patient as I have been doing (to maintain the status quo) |  |  |  |
| …to justify the way I interact with the patient (to resolve a doubt) |  |  |  |
| …to change the way I interact with the patient (to modify my methods) |  |  |  |
| …to persuade the patient or other health professionals to make changes |  |  |  |
| **Do you expect your patient’s health to be improved as a result of using this information?** | **Yes** | **Maybe** | **No** |
| I do not expect that the information will improve my patient’s health |  |  |  |
| I expect that the information will… |  |  |  |
| …make the patient more knowledgeable about health or healthcare |  |  |  |
| …increase the patient’s satisfaction with a treatment, a diagnostic test or a preventive intervention |  |  |  |
| …avoid a useless or harmful treatment, diagnostic test or preventive intervention |  |  |  |
| …prevent an illness, a deterioration in health, or an acute episode of a chronic illness |  |  |  |
| …improve the patient’s health, autonomy or resilience (that is,the patient’s capacity to handle adversity) |  |  |  |

1. **Please circle the answer of your choice:**
2. I intend to use in my practice what I learned from the document to help my patients make an informed decision.

| **-3** | **-2** | **-1** | **0** | | **+1** | **+2** | **+3** |
| --- | --- | --- | --- | --- | --- | --- | --- |
| **Strongly disagree** | | | | **Strongly agree** | | | |

# Most of my patients would approve/disapprove of my using in my practice what I learned from the document to help them make an informed decision.

| **-3** | **-2** | **-1** | **0** | | **+1** | **+2** | **+3** |
| --- | --- | --- | --- | --- | --- | --- | --- |
| **Would strongly disapprove** | | | | **Would strongly approve** | | | |

1. Using in my practice what I learned from this document to help a patient make a decision seems to me to be...

| **a)** | | -3 | -2 | -1 | 0 | | 1 | 2 | 3 | | --- | --- | --- | --- | --- | --- | --- | --- | | Very useless | | | | Very useful | | | | |
| --- | --- | --- | --- | --- | --- | --- | --- | --- | --- | --- | --- | --- | --- | --- | --- | --- | --- |
|  |  |
| **b)** | | -3 | -2 | -1 | 0 | | 1 | 2 | 3 | | --- | --- | --- | --- | --- | --- | --- | --- | | Very difficult | | | | Very easy | | | | |
|  |  |
| **d)** | | -3 | -2 | -1 | 0 | | 1 | 2 | 3 | | --- | --- | --- | --- | --- | --- | --- | --- | | Very irresponsible | | | | Very responsible | | | | |
|  |  |
| **f)** | | -3 | -2 | -1 | 0 | | 1 | 2 | 3 | | --- | --- | --- | --- | --- | --- | --- | --- | | Very unlikely | | | | Very likely | | | | |
|  |  |
| **h)** | | -3 | -2 | -1 | 0 | | 1 | 2 | 3 | | --- | --- | --- | --- | --- | --- | --- | --- | | Very harmful | | | | Very beneficial | | | | |

# Most people in my professional surroundings would approve/disapprove of my using in my practice what I learned from the document to help my patients make an informed decision.

| **-3** | **-2** | **-1** | **0** | | **+1** | **+2** | **+3** |
| --- | --- | --- | --- | --- | --- | --- | --- |
| **Would strongly disapprove** | | | | **Would strongly approve** | | | |

1. I feel able to use in my practice what I learned from this document to help my patients make an informed decision.

| **-3** | **-2** | **-1** | **0** | | **+1** | **+2** | **+3** |
| --- | --- | --- | --- | --- | --- | --- | --- |
| **Strongly disagree** | | | | **Strongly agree** | | | |

# It is likely/unlikely that I will use in my practice what I learned from the document to help my patients make an information decision.

| **-3** | **-2** | **-1** | **0** | | **+1** | **+2** | **+3** |
| --- | --- | --- | --- | --- | --- | --- | --- |
| **Very unlikely** | | | | **Very likely** | | | |

1. I see no reason why not to use in my practice what I learned from this document to help my patients make an informed decision.

| **-3** | **-2** | **-1** | **0** | | **+1** | **+2** | **+3** |
| --- | --- | --- | --- | --- | --- | --- | --- |
| **Strongly disagree** | | | | **Strongly agree** | | | |

1. People who are important to me would urge me to use in my practice what I learned from this document to help my patients make an informed decision.

| **-3** | **-2** | **-1** | **0** | | **+1** | **+2** | **+3** |
| --- | --- | --- | --- | --- | --- | --- | --- |
| **Strongly disagree** | | | | **Strongly agree** | | | |

# This document will help me communicate information that the patient needs to make a decision.

| **-3** | **-2** | **-1** | **0** | | **+1** | **+2** | **+3** |
| --- | --- | --- | --- | --- | --- | --- | --- |
| **Strongly disagree** | | | | **Strongly agree** | | | |

# This document does more to improve my clinical knowledge that do other documents of the kind.

| **-3** | **-2** | **-1** | **0** | | **+1** | **+2** | **+3** |
| --- | --- | --- | --- | --- | --- | --- | --- |
| **Strongly disagree** | | | | **Strongly agree** | | | |

# This document will do more to improve my clinical practices than would other documents of the kind.

| **-3** | **-2** | **-1** | **0** | | **+1** | **+2** | **+3** |
| --- | --- | --- | --- | --- | --- | --- | --- |
| **Strongly disagree** | | | | **Strongly agree** | | | |

# This document will help my clinical work.

| **-3** | **-2** | **-1** | **0** | | **+1** | **+2** | **+3** |
| --- | --- | --- | --- | --- | --- | --- | --- |
| **Strongly disagree** | | | | **Strongly agree** | | | |

# This document is easy to use.

| **-3** | **-2** | **-1** | **0** | | **+1** | **+2** | **+3** |
| --- | --- | --- | --- | --- | --- | --- | --- |
| **Strongly disagree** | | | | **Strongly agree** | | | |

# This document makes it easy for me to find the information the patient needs to make a decision.

| **-3** | **-2** | **-1** | **0** | | **+1** | **+2** | **+3** |
| --- | --- | --- | --- | --- | --- | --- | --- |
| **Strongly disagree** | | | | **Strongly agree** | | | |

# It will be easy for me to use what I learned from this document to help my patients make an informed decision.

| **-3** | **-2** | **-1** | **0** | | **+1** | **+2** | **+3** |
| --- | --- | --- | --- | --- | --- | --- | --- |
| **Strongly disagree** | | | | **Strongly agree** | | | |

# I find it easy to understand the information presented in this document.

| **-3** | **-2** | **-1** | **0** | | **+1** | **+2** | **+3** |
| --- | --- | --- | --- | --- | --- | --- | --- |
| **Strongly disagree** | | | | **Strongly agree** | | | |

***Thank you for having completed this questionnaire***
